# Durable CRISPR-Based Epigenetic Silencing

**DOI:** 10.34133/2021/9815820

**Published:** 2021-06-30

**Authors:** Muneaki Nakamura, Alexis E. Ivec, Yuchen Gao, Lei S. Qi

**Affiliations:** ^1^Department of Bioengineering, Stanford, CA 94305USA; ^2^Program in Human Biology, Stanford, CA 94305USA; ^3^Cancer Biology Program, Stanford, CA 94305USA; ^4^Department of Chemical and Systems Biology, Stanford, CA 94305USA; ^5^ChEM-H Institute, Stanford, CA 94305USA

## Abstract

Development of CRISPR-based epigenome editing tools is important for the study and engineering of biological behavior. Here, we describe the design of a reporter system for quantifying the ability of CRISPR epigenome editors to produce a stable gene repression. We characterize the dynamics of durable gene silencing and reactivation, as well as the induced epigenetic changes of this system. We report the creation of single-protein CRISPR constructs bearing combinations of three epigenetic editing domains, termed KAL, that can stably repress the gene expression. This system should allow for the development of novel epigenome editing tools which will be useful in a wide array of biological research and engineering applications.

## 1. Introduction

The adoption of CRISPR systems has enabled a wide range of synthetic biology applications by allowing the rapid targeting of CRISPR-associated (Cas) proteins to almost anywhere in the genome via a guide RNA (gRNA). In particular, nuclease-deactivated Cas (dCas) proteins have been applied for use of controlling the gene expression via directed dCas binding [1] and the localization of domains driving gene up- and downregulation (termed CRISPRa and CRISPRi, respectively) [2, 3]. Further studies have identified improved domains (or combinations thereof) for enhanced activation [4, 5] or repression [6, 7] to increase the utility of these technologies. A typical feature of CRISPRi/a technologies is that gene expression changes are maintained only while the effector domains remain actively targeted to the locus of interest [8]. Although this may be beneficial in many contexts, in other applications, it may be desirable to generate gene regulation changes that can persist after a transient dose of the CRISPR effector.

The cooption of epigenetic processes has long been examined as a possible route for inducing long-lasting gene expression changes [9], with much research in particular focusing on DNA CpG methylation as a mark highly associated with persistent gene silencing. However, the gene expression changes induced by engineered DNA methylation remain quite modest. A report describing the simultaneous use of the Krüppel-associated box (KRAB) domain commonly used in CRISPRi along with DNMT3A and DNMT3L domains (involved in *de novo* DNA methylation) showed significant, long-lasting gene repression [10]. This study, along with others using the same combination of domains [11, 12], however, demonstrated mixed repression capability depending on the gene target, raising the question of the context dependence of this approach. To address this issue, we adopted a synthetic biology approach, creating a synthetic reporter system in order to systematically and quantitatively assess the activity of domains fused to dCas to induce stable gene silencing. We used this standardized synthetic biology platform to further understand the underlying contributors toward gene silencing and to engineer new CRISPR epigenetic effectors for modulating gene expression.

## 2. Materials and Methods

### 2.1. Experimental and Technical Design

**Figure 1:**
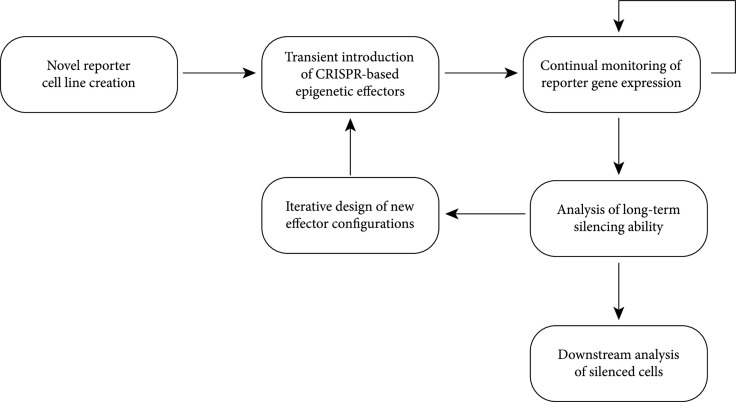
Overview of experimental scheme.

To enable this controlled environment for testing, (Figure 1) we first created a new standardized reporter cell line that could be used to quantitatively compare the activity of CRISPR-based effectors. Subsequently, we transiently introduced CRISPR effectors into this reporter line and monitored the resulting change on the reporter gene expression over time. Analysis of these results informed the design of next-generation effector combinations in an iterative design cycle. The silenced cells were also subjected to further characterization to better understand the nature of the gene silencing.

### 2.2. Cell Culture

HEK293T cells were cultured in DMEM containing sodium pyruvate, GlutaMAX, and 4.5 g/L glucose (Thermo Fisher Scientific) supplemented with 10% FBS. Cells were not tested for mycoplasma contamination.

HEK293T cells bearing EGFP expressed from the SV40 promoter, used in previous studies [8, 13], were lentivirally transduced with a cassette expressing gRNA targeting the SV40 promoter and BFP and puromycin resistance proteins. Selection with 2 *μ*g/mL puromycin resulted in near purity of cells bearing both cassettes.

### 2.3. Flow Cytometry Analysis

For time-course experiments, plasmids bearing *Streptococus pyogenes* (*Spy*) dCas9 effector fusions and Zeocin resistance markers were transfected into reporter cells in 12-well plates using TransIT-LT1 reagent (Mirus), with 1 *μ*g DNA split evenly among plasmids used in each transfection. Cells were exposed to 400 *μ*g/mL Zeocin (Thermo Fisher Scientific) days 2-5 posttransfection. Cells were serially passaged and assessed for fluorescence via flow cytometry with a CytoFLEX S machine (Beckman Coulter).

Reactivation experiments were performed in stably silenced cell populations. Transient reactivation was assessed at day 5 using effectors bearing mCherry fluorescent markers and gating for mCherry-positive cells.

### 2.4. Epigenetic Characterization

Bisulfite conversion (Zymo) of genomic DNA from silenced and untreated cells was performed and transformed into *E. coli* using a TOPO Blunt kit (Thermo Fisher Scientific). DNA from individual colonies was PCR-amplified and submitted for Sanger sequencing.

For ChIP-qPCR experiments, untreated reporter cells and silenced cells (additionally sorted for negative GFP expression) were fixed in formaldehyde (Thermo Scientific). The reaction was quenched with glycine, and the cells were washed with solution containing 0.5% Igepal and 1 mM PMSF. Cell pellets were flash-frozen in liquid nitrogen and submitted for downstream processing (Active Motif).

### 2.5. Data Analysis

Raw flow cytometry data were compensated and analyzed using FlowJo software, gating for live singlet cells. The resulting data were processed and analyzed using custom scripts.

The data were normalized at each time point to the median value of unsilenced cells. Time courses of median fluorescence of each condition were fit using least-squares to an asymmetric Gaussian of the following form:

(1)It=I0−Ae−k1t−τ2,t<τ,If−If−I0+Ae−k2t−τ2,t≥τ,

with I indicating median fluorescent intensity. $I_{0}$ was parameterized by the fluorescence level of untransfected reporter cells, and all other parameters were varied to fit the model. Fold-change in intensity was calculated by the ratio $I_{0}/I_{f}$. For temporary repression, maximal fold-change was determined from the lowest expression value measured throughout a given time course.

For population-level analysis, GFP-negative fluorescence threshold was set at the 99th percentile of cells lacking reporter cassette. Stably silenced populations (defined as cell populations>21 days posttransfection) were used to calculate GFP-negative proportion; such proportions were calculated for each time point and experiment and averaged to obtain the value for each condition. These stably silenced populations were pooled and were fit to a sum of three Gaussians. The amplitude and mean were allowed to vary, along with a Gaussian width parameter (shared among the three Gaussians).

## 3. Results

### 3.1. Characterization of dCas9-Based Gene Silencing on a Standard Fluorescent Reporter System

**Figure 2:**
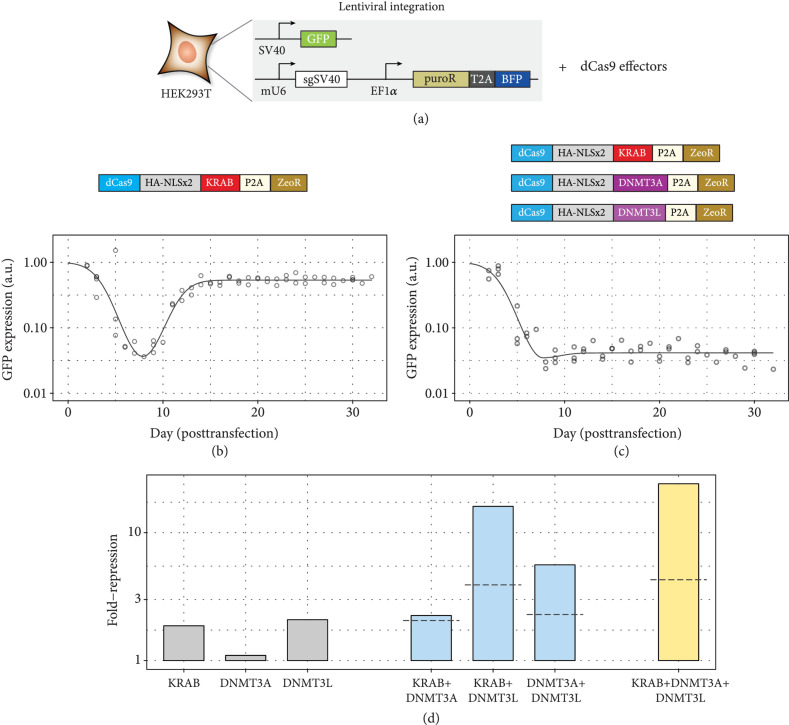
Assay for assessing long-term gene silencing. (a) A fluorescent GFP reporter driven by the SV40 promoter is integrated into HEK293T cells, along with a cassette that guides dCas9 to bind to the SV40 promoter. These cells can be transiently transfected with dCas9 fused to various domains to assess the ability to repress GFP over time. (b, c) GFP expression over time following transfection of effectors. Dots indicate individual time points, and model fit is indicated by solid lines. (b) Transfection of dCas9-KRAB. (c) Cotransfection of dCas9-KRAB, dCas9-DNMT3A, and dCas9-DNMT3L. (d) Long-term silencing ability of various effectors individually (gray) or in combinations of two domains (blue) or three (yellow) domains. Dashed lines indicate predicted value if the relevant domains’ silencing activity adds independently.

We based our reporter on a cassette containing GFP driven by the constitutive SV40 promoter, along with a corresponding cassette expressing the relevant gRNA targeting the GFP promoter. These constructs were lentivirally introduced into HEK293T cells. The resultant cells contain all components except the relevant CRISPR protein effectors, allowing the transient introduction of effectors to assess the ability to drive GFP silencing under a consistent cellular environment (Figure 2(a), Supp. Fig. 1a, b).

We created a set of constructs each bearing one of the three relevant domains (KRAB, DNMT3A, and DNMT3L) fused to *Spy* dCas9 protein and additionally expressing a separate Zeocin resistance domain via the self-cleaving P2A peptide tag (Figures 2(b) and 2(c)). Reporter cells were transiently transfected with these constructs, subjected to Zeocin selection to enrich for successful introduction of the transfected construct, and the fluorescence monitored over time via flow cytometry.

Consistent with prior studies [2, 8, 13], we observed significant GFP repression with dCas9-KRAB shortly following transfection and selection, with subsequent recovery of expression at long timescales (Figure 2(b), Supp. Fig. 1c). Conversely, with cotransfection of all three dCas9 effectors, we saw the formation of a population of cells demonstrating repression of GFP expression that was stable for weeks posttransfection (Figure 2(c), Supp. Fig. 1d).

We assessed the relative contributions of each domain to the silencing process by transfecting each of the domains individually or in pairwise combinations (Figure 2(d), Supp. Fig. 2). We observed that the domains individually exhibited only minor ability to generate stably silenced cells. Interestingly, dCas9-DNMT3A (containing the active catalytic methyltransferase domain within the DNMT3A:DNMT3L complex) exhibited the lowest silencing activity, while dCas9-DNMT3L was the most active.

This role of DNMT3L was emphasized in the dual-effector combination results. Although dCas9-KRAB+dCas9-DNMT3A in combination did not appreciably increase the silencing efficiency relative to dCas9-KRAB, dCas9-DNMT3L significantly enhanced the proportion of silenced cells and increased the long-term repression of reporter expression when combined with either of the other domains. This synergy with the other domains was superadditive, with the addition of the third domain providing further synergy (Figure 2(d), Supp. Fig. 2f). These results may indicate a key role of DNMT3L in inducing silencing, at least in the context of CRISPR protein fusions and HEK293T cells.

### 3.2. Characterization of Silenced Cells and Reactivation

**Figure 3:**
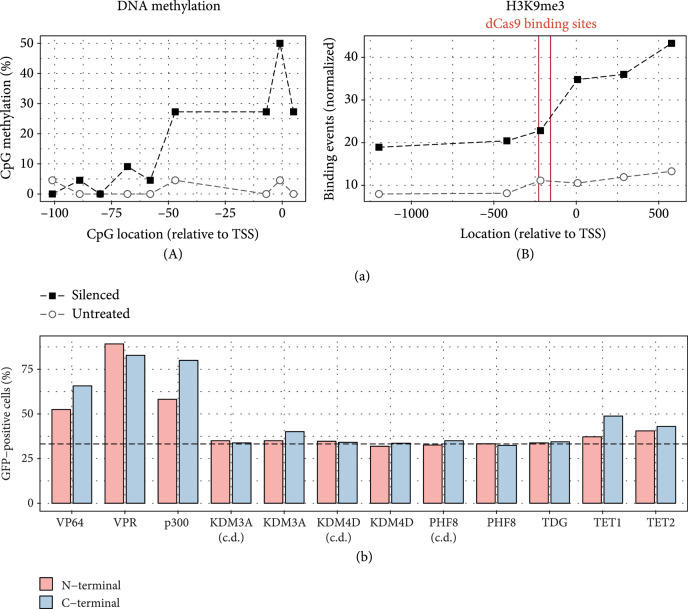
Characterization of silenced cells. (a) Assessment of DNA methylation (A) and H3K9me3 (B) of cells exposed to dCas9-KRAB+dCas9-DNMT3A+dCas9-DNMT3L versus untreated cells near reporter promoter. Binding sites of dCas9 are indicated by red lines. (b) Reactivation of silenced cells using various domains fused to the N-terminus (pink) or C-terminus (blue) of dCas9 measured at 5 days posttransfection. Dotted line indicates the GFP-positive value of untreated cells.

Subsequent examination of the targeted reporter region in silenced cells revealed epigenetic changes (CpG methylation and H3K9me3) associated with gene repression that extended up to hundreds of base pairs away from the dCas9 binding site (Figure 3(a)). We also assessed the ability of the silenced cells to be reactivated. Transient activity of various activation and epigenetic remodeling domains at five days posttransfection revealed that previously described temporary activators demonstrated the strongest reactivation potential (Figure 3(b)), with negligible impact from most other domains except for a small effect from the TET1 and TET2 domains (involved in accelerating DNA demethylation). Time courses using a similar Zeocin pulse assay showed that CRISPRa-mediated reactivation was indeed transient, while TET1 and TET2 could maintain stable reactivation over time (Supp. Fig. 3).

### 3.3. Analysis of Novel Domain Configurations for Gene Silencing

We next applied our reporter system to the analysis and engineering of new domain configurations, examining first the possibility of condensing multiple epigenetic effectors onto a single dCas9 protein. Condensing multiple effectors to a single dCas9 fusion protein may be beneficial for gene silencing activity. By incorporating multiple domains, it is possible to introduce the requisite effector cocktail using fewer plasmids, which likely simplifies delivery and also increases the likelihood of selection enriching for cells containing all epigenetic editing domains. Furthermore, with fewer dCas9 effectors, there may be less competition to shared binding sites encoded by a single gRNA. Conversely, more fused domains result in longer proteins and may impair full expression and folding, as well as introduce additional steric hindrances.

**Figure 4:**
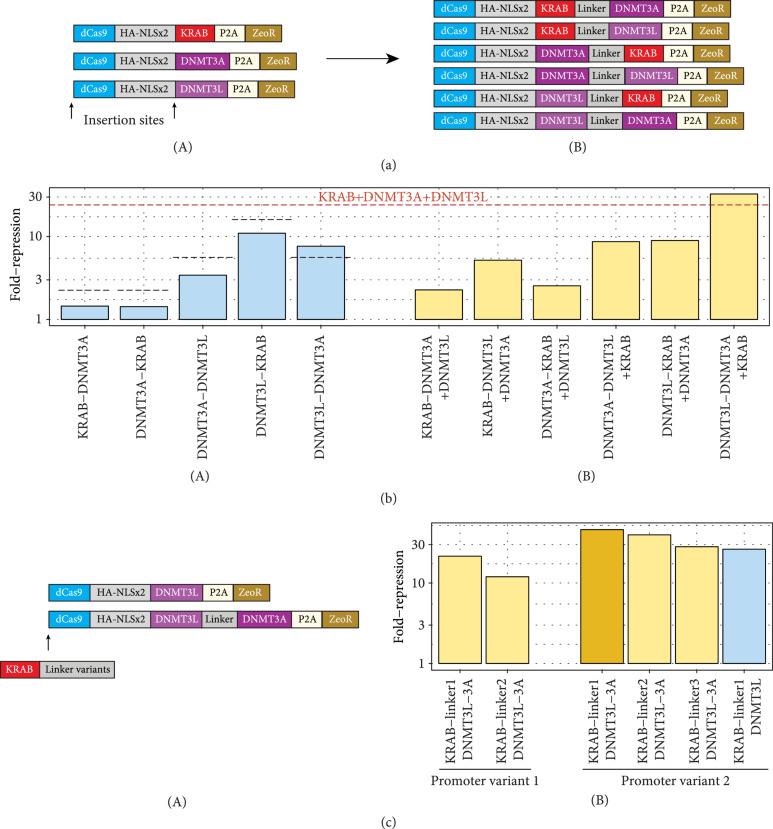
Assessment of combinatorial configurations for gene silencing. (a) Scheme depicting the insertion of combinations of effector domains. (A) Combinatorial dual-effector constructs. (b) Summary of silencing capabilities of dual-effector constructs alone (A, blue) and in combination with cognate third effector (B, yellow). (A) Dotted lines show experimental silencing values of the relevant two domains on separate dCas9s. Dashed red line indicates silencing value of using all three domains on separate dCas9s. (c) (A) Depiction of strategy to introduce KRAB at N-terminus of dCas9 to create all-in-one constructs. (B) Silencing activity of all-in-one constructs.

To investigate the relative contribution of these effects we moved from a three-plasmid system to a two-plasmid system, creating combinatorial dual-domain variants fused serially at the C-terminus of dCas9 (Figure 4(a)). We found that the silencing capabilities of these constructs varied widely, with those containing DNMT3L being the most active (Figure 4(b), Supp. Fig. 4). Of note, most dual-effector constructs showed lower silencing ability than the corresponding effectors transfected individually as two plasmids, with the exception of dCas9-DNMT3L-DNMT3A.

These results were corroborated in a series of experiments wherein we cotransfected each dual-effector construct with the cognate effector plasmid to reconstitute the triple-effector cocktail (Figure 4(b), Supp. Fig. 5). Again, all such combinations with dual-effector constructs demonstrated impaired silencing activity relative to the triple-plasmid system, except for the dCas9-DNMT3L-DNMT3A+dCas9-KRAB condition. Based on these results, we infer that the DNMT3L-DNMT3A fusion likely recapitulates the synergistic effects of the individual dCas9-DNMT3L and dCas9-DNMT3A constructs.

### 3.4. Interaction of Silencing Constructs with Other Epigenetic Domains

Having created separate CRISPR effectors encoding transcriptional repression (KRAB) and DNA methylation (DNMT3L-DNMT3A), we next investigated the modularity of these domains in inducing gene silencing. We first tested repressor domains (SID [14] and ZIM3 KRAB [15]) that were reported to have better silencing performance than the ZNF10 KRAB typically used for CRISPRi applications (Supp. Fig. 6). In this assay, SID demonstrated only small amounts of temporary repression, while ZIM3 KRAB did produce approximately 2-fold greater maximal repression compared to ZNF10 KRAB (Supp. Fig. 6g). Comparing the ability to stably silence cells, we did not observe an appreciable difference between ZNF10 and ZIM3 KRAB domains, with or without dCas9-DNMT3L-DNMT3A, while dCas9-SID+dCas9-DNMT3L-DNMT3A produced less silencing than dCas9-DNMT3L-DNMT3A alone.

We also tested the ability of a prokaryotic DNA methyltransferase, the MQ1 domain from *M. SssI* [16–18], to induce silencing with and without dCas9-KRAB but observed no additional silencing effect associated with this domain (Supp. Fig. 7). These results in total suggest that KRAB+DNMT3A/DNMT3L is a particularly potent epigenetic combination that is not easily replicable by swapping other domains and that this synergy with DNMT3L-DNMT3A may be generalizable to other KRAB domains.

### 3.5. Creation of All-in-One CRISPR Silencers (dCas9-KAL)

We next investigated whether we could combine the prior results to further condense all necessary domains into a single dCas9 construct. Since all combinations of KRAB with DNMT3A or DNMT3L demonstrated impaired silencing performance relative to the domains by themselves, we hypothesized that unfavorable steric hindrances between the KAP1 complex recruited by KRAB and the DNMT3A:DNMT3L complex may have prevented full activity of the effector domains. Therefore, we used our most active dCas9-DNMT3L-DNMT3A construct and tested N-terminal fusions of the KRAB domain, terming this combination of effectors KAL (**K**RAB-DNMT3**A**-DNMT3**L**). We varied the linker lengths bridging the KRAB and dCas9, ranging from 12 amino acids (aa) (linker1), 27 aa [19] (linker2), and 147 aa [20, 21] (linker3) in size, as well as the promoter sequence (Figure 4(c), Supp. Fig. 8).

We observed that all KAL combinations were capable of inducing high levels of stable gene repression. Interestingly, there seemed to be an inverse correlation of silencing activity and linker length, implying that repositioning the KRAB domain to the N-terminus was sufficient to constitute an active triple-effector. This was corroborated by creating a dual-effector construct with an N-terminal KRAB and C-terminal DNMT3L domain (KL). dCas9-KL also strongly silenced gene expression (Figure 4(c), Supp. Fig. 8d), indicating that direct fusion of DNMT3A is dispensable for durable gene repression.

We next examined the population distributions of silenced cells. Plotting the relationship between long-term fold-change repression and percentage of silenced cells, we observed a strong correlation between the two metrics (Supp. Fig. 9a), implying that the two are related processes. To investigate this in more detail, we fit the long-term populations to a sum of Gaussian distribution (Supp. Fig. 9b-d), separating each condition into low-, medium-, and high-expressing populations. This revealed that there was an enrichment in the proportion residing in the lowest-expressing population that increased with more efficient silencers and that this was also associated with a reduction in expression across all conditions, suggesting that effective epigenetic silencing may be derived from both conversion efficiency into the most silenced cell population as well as reduction in gene expression across the whole population.

## 4. Discussion

The development of new epigenome editing tools for gene regulation is greatly assisted by synthetic biology assays to quantitatively measure their effects. Our system here enables a simple assay for the distinguishing of domain fusions to dCas9 on the single-cell level without the need for sorting. Using this system, we were able to find new domain combinations (KAL and KL) that combine all effectors necessary to induce gene silencing on a single protein. We hypothesize that such a CRISPR construct may be useful in applying epigenetic-based repression to other biological contexts.

One aspect revealed by our study is the dependence of the silencing activity on the relative configuration of effector domains. The better performance of our DNMT3L-DNMT3A fusion relative to the DNMT3A-DNMT3L construct might result from the distances of the N- and C-termini of the DNMT3A and DNMT3L domains when assembled into the active heterotetramer complex [22, 23]. A previously described single-protein fusion of DNMT3A-DNMT3L [12, 19, 24–27] used a 27 aa linker, and the impaired activity of our DNMT3A-DNMT3L (using a 12 aa linker) demonstrates that a longer linker may be necessary. Conversely, we were able to compensate for this by swapping the orientation of the domains.

Similarly, separating the KRAB domain was critical for obtaining maximal silencing activity. However, more separation was not necessarily better: for our constructs, shorter linkers demonstrated greater gene silencing. Another recent study [27] also showed that separating DNMT3A-DNMT3L and KRAB showed high silencing activity. In sum, these results highlight the importance of linker and fusion optimization to obtain the highest CRISPR-based activity.

Our results also suggest that there is some inherent synergy between the KRAB and DNMT3A : DNMT3L complexes. A study reported that EZH2 in combination with DNMT3A-DNMT3L was able to generate stable silencing in certain gene and cellular contexts [12], but we were unable to generate stable silencing with other epigenetic domains outside of these systems. We are unable to rule out effects from positioning (such as smaller methylation window from the MQ1 domain [16]), but this may also be derived from differences in epigenetic changes generated [28] or interaction partners within the cell.

Although we show that silencing can be achieved (in combination with DNMT3L-DNMT3A) across different KRAB domains, greater KRAB repressive ability per se did not seem to measurably enhance long-term silencing activity, perhaps suggesting that synergy may be saturated or operates via a different mechanism. Whether KRAB domains incorporating other epigenetic domains [6] or mutations to enhance activity or epigenetic memory [7] can increase combinatorial silencing ability remains to be determined.

We also find that the ability of domains in silencing gene expression is a continuous, rather than binary phenomenon. Our results are consistent with a prior report that implied a two-state reporter system that can be turned off using KRAB or DNMT3A alone, albeit with lower efficiency [29]. However, our results also suggest that there may be more stable states than simply on or off, which bears further investigation. We also report that KRAB+DNMT3L is sufficient to efficiently silence gene expression. A prior report found that dCas9-KRAB along with free DNMT3L overexpression also could stably repress genes [12], but to our knowledge, this is the first report of a single protein containing these two domains applied to gene silencing.

The synthetic biology approach described in this work allows for the rapid determination of silencing capabilities. By making quantitative measurements of gene expression changes, it should be possible to stringently test novel repression domains and configurations thereof, which will provide enhanced tools for generating long-lasting gene expression changes within cells.

## Supplementary Materials

Supplementary Materials Supplementary Figure 1: assessment of the reporter system. Supplementary Figure 2: silencing of single and combinations of effectors. Supplementary Figure 3: reactivation of silenced cells. Supplementary Figure 4: dual-effector construct silencing. Supplementary Figure 5: combinations of double-effector and single-effector constructs. Supplementary Figure 6: characterization of temporary repressor variants. Supplementary Figure 7: characterization of dCas9-MQ1 silencing. Supplementary Figure 8: characterization of KAL and KL effectors. Supplementary Figure 9: population characterization of gene silencing.
