# Supplementary material for: Durable CRISPR-Based Epigenetic Silencing
Source: Biodes Res. 2021 Jun 30;2021:9815820. doi: 10.34133/2021/9815820 (PMC10521745; doi:10.34133/2021/9815820)
Supplement: Supplementary Materials — Supplementary Figure 1: assessment of the reporter system. Supplementary Figure 2: silencing of single and combinations of effectors. Supplementary Figure 3: reactivation of silenced cells. Supplementary Figure 4: dual-effector construct silencing. Supplementary Figure 5: combinations of double-effector and single-effector constructs. Supplementary Figure 6: characterization of temporary repressor variants. Supplementary Figure 7: characterization of dCas9-MQ1 silencing. Supplementary Figure 8: characterization of KAL and KL effectors. Supplementary Figure 9: population characterization of gene silencing. [file 9815820.f1.docx]

**Supplementary Materials**

*Durable CRISPR-based epigenetic silencing*

Muneaki Nakamura, Alexis E. Ivec, Yuchen Gao, Lei S. Qi

**Supplementary Figures**


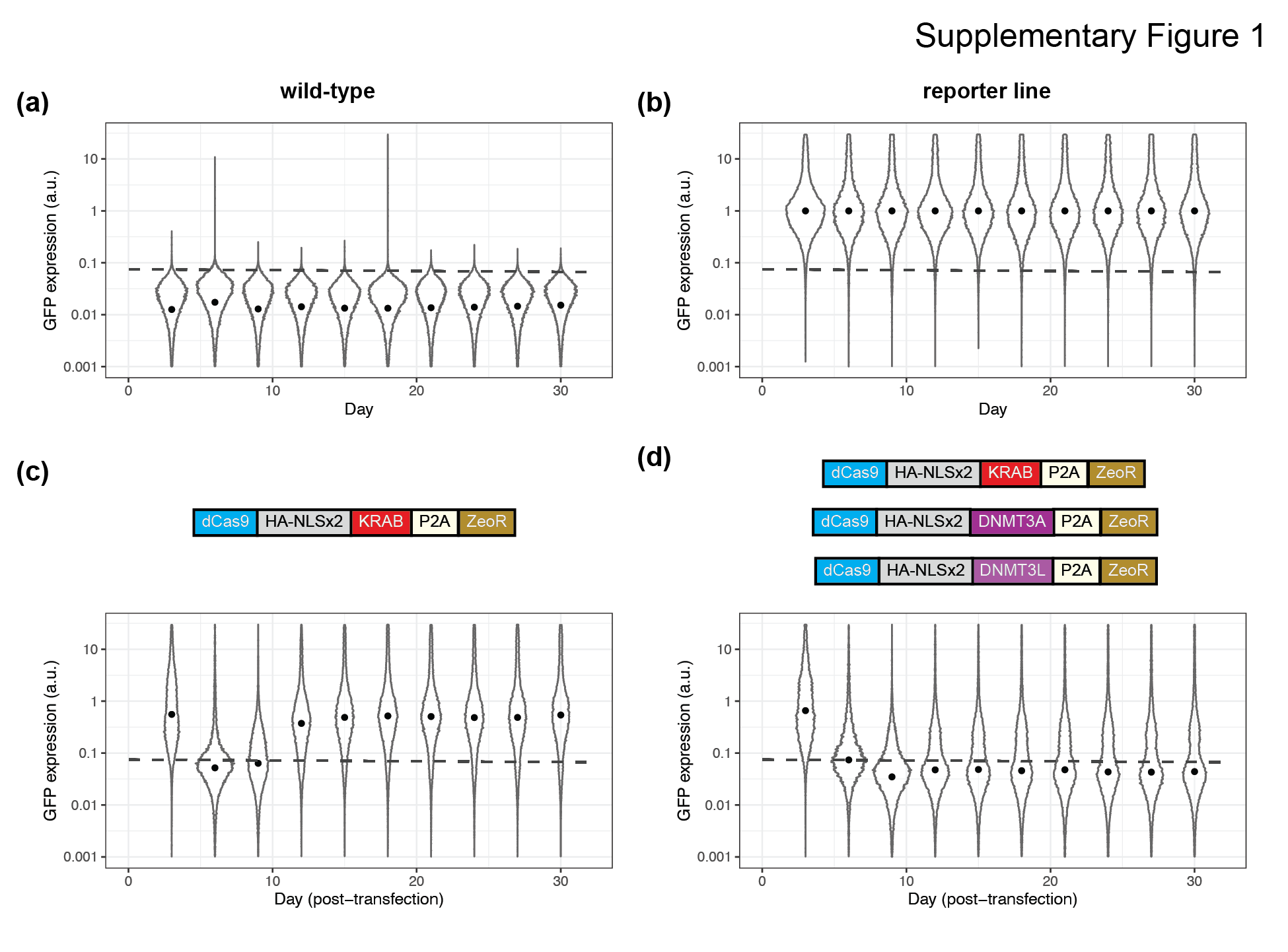


**Supplementary Figure 1 – assessment of reporter system**

**(a-d)** Representative GFP fluorescence distributions over time plotted as violin plots. Median fluorescence indicated by black dots. Dashed line indicates approximate cutoff of no fluorescence, as determined by wild-type HEK293T cells. **(a-b)** Traces of cell lines untransfected and not exposed to Zeocin. **(a)** Wild-type HEK293T cell line without integrated reporter. **(b)** Reporter HEK293T cell line with GFP and sgRNA cassette integrated. **(c-d)** Traces of reporter cell line following transfection of indicated dCas9 effector plasmid and Zeocin selection. **(c)** Transfection of dCas9-KRAB alone. **(d)** Co-transfection of dCas9-KRAB, dCas9-DNMT3A, and dCas9-DNMT3L plasmids.


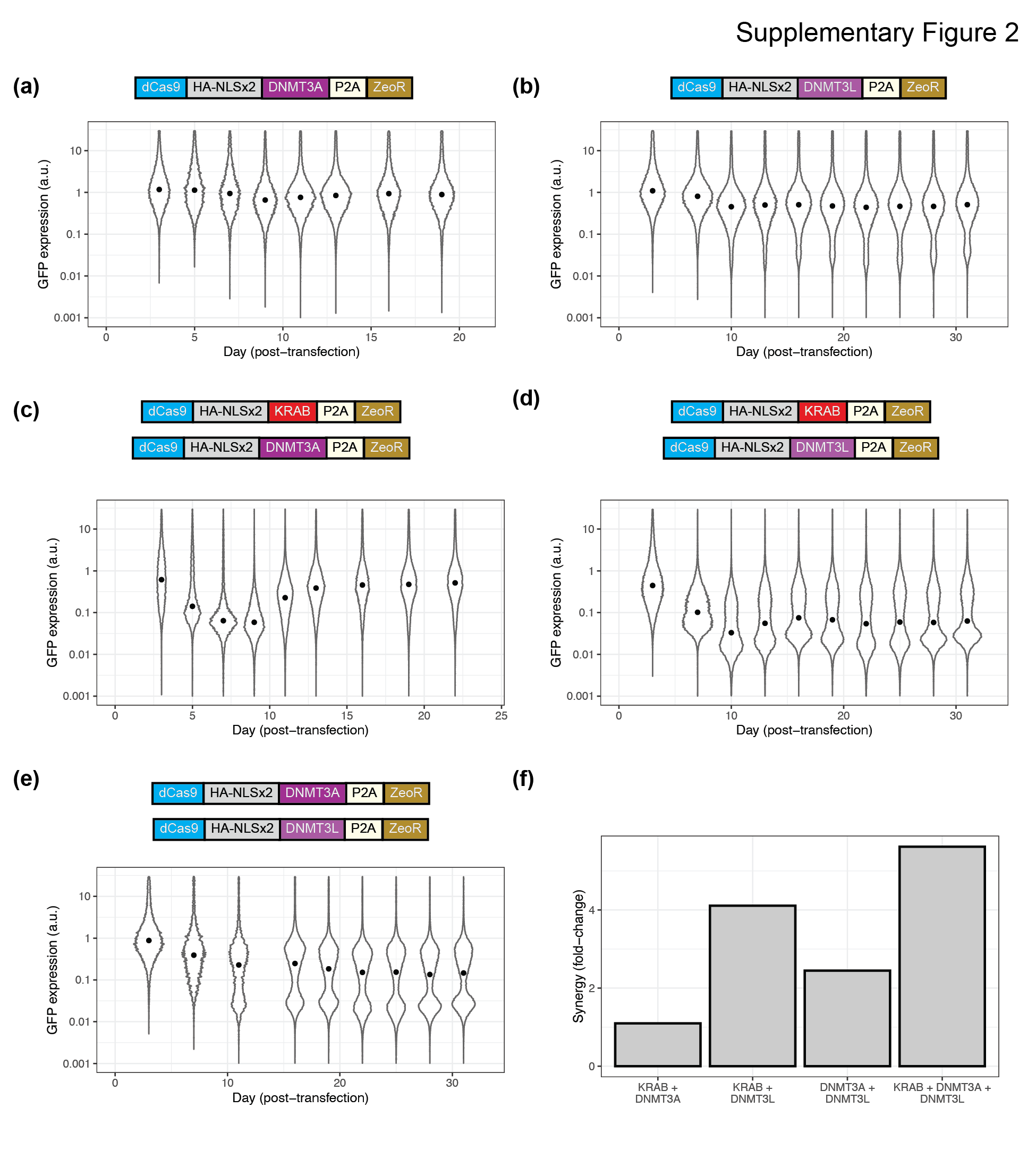


**Supplementary Figure 2 – silencing of single and combinations of effectors**

**(a-e)** Representative violin plots of raw GFP fluorescence distributions over time following transient transfection of effector and Zeocin selection. Median fluorescence indicated by black dots. **(a)** Transfection of dCas9-DNMT3A. **(b)** Transfection of dCas9-DNMT3L. **(c)** Co-transfection of dCas9-KRAB and dCas9-DNMT3A. **(d)** Co-transfection of dCas9-KRAB and dCas9-DNMT3L **(e)** Co-transfection of dCas9-DNMT3A and dCas9-DNMT3L **(f)** Assessment of synergy of combinations of effectors. For each combination, a calculated value of the expected long-term repression if the respective individual domains' repression fold-changes multiplied independently was determined. The synergy value is the experimental long-term fold-repression from the combination of effectors divided by the calculated value.

**
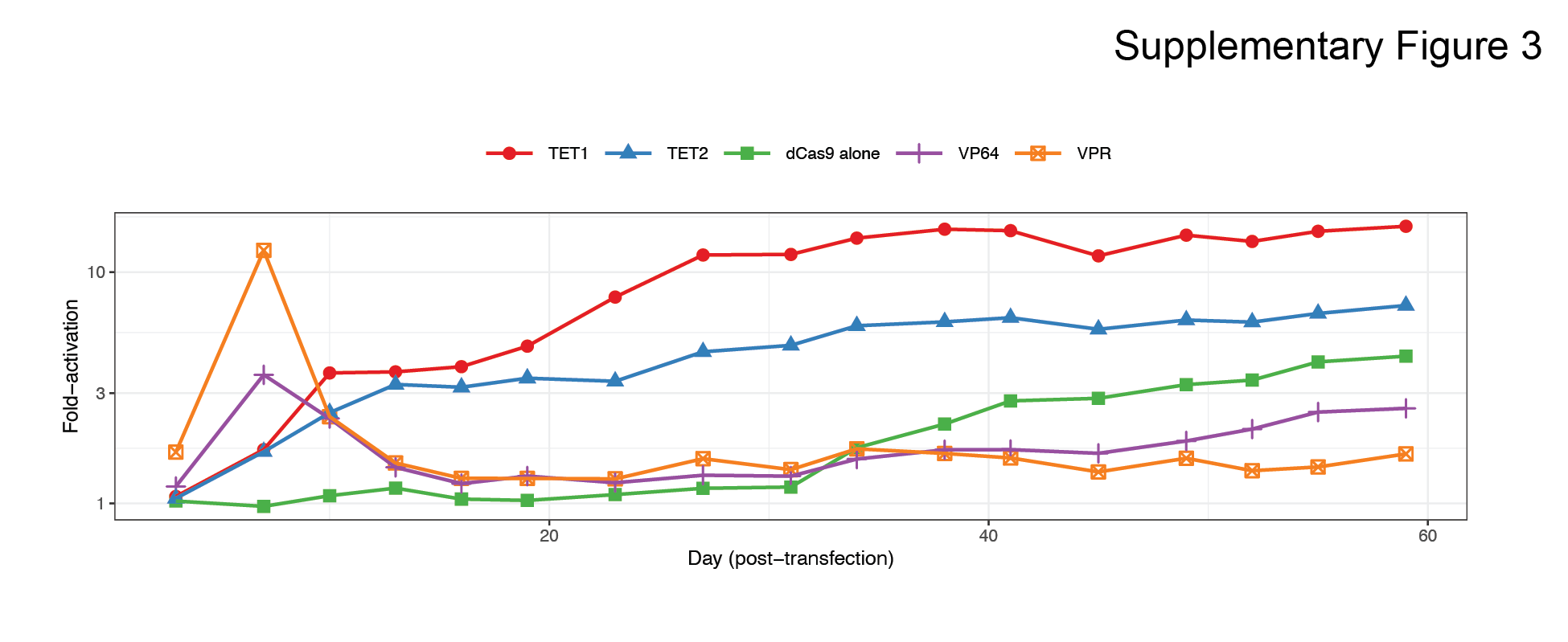
**

**Supplementary Figure 3 – reactivation of silenced cells**

Timecourse of reactivation of silenced cells from various dCas9 effectors. Fold-activation is calculated relative to a mock transfection control.


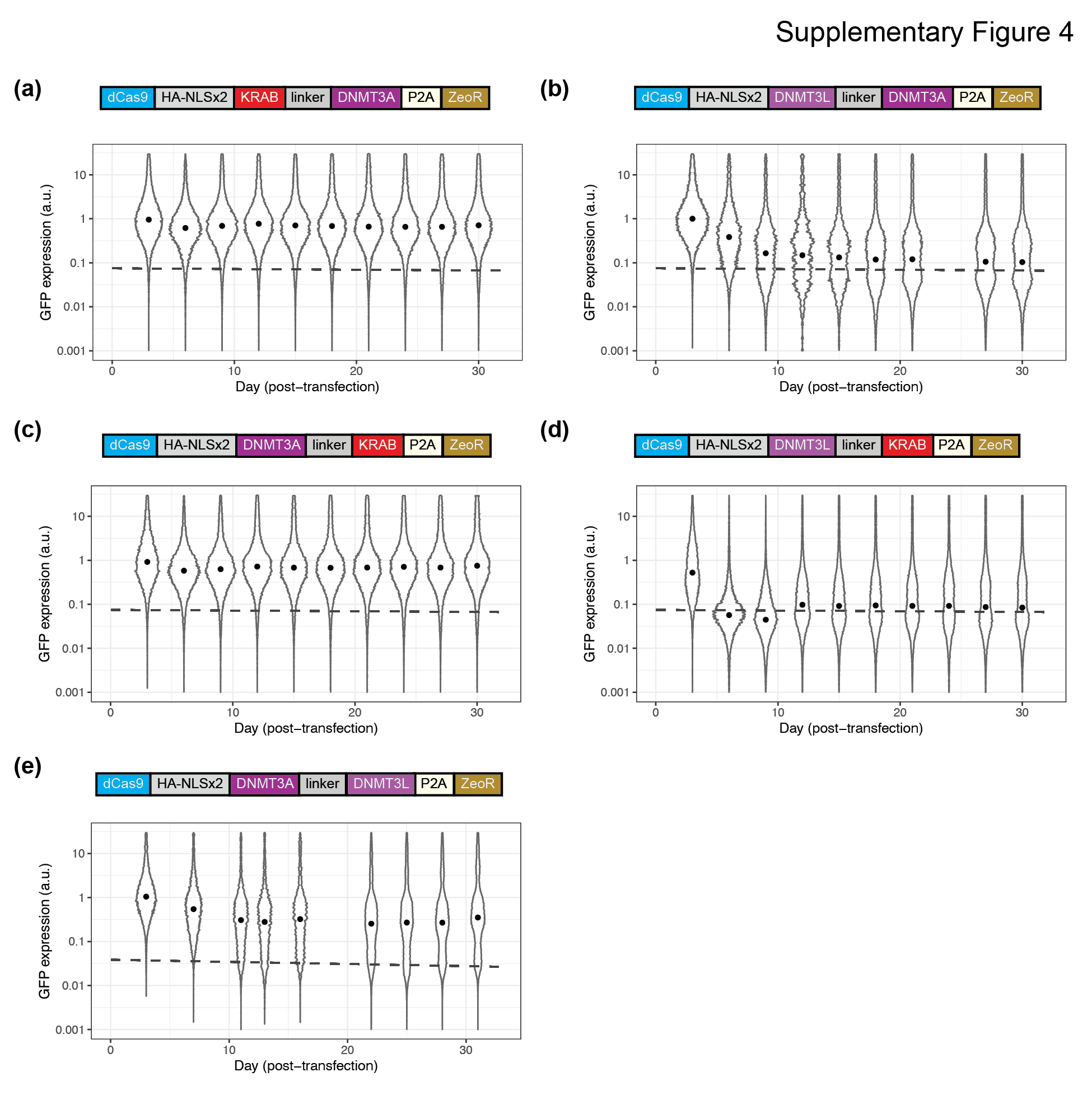


**Supplementary Figure 4 – dual-effector construct silencing**

Representative violin plots of raw GFP fluorescence distributions over time following transient transfection of effector and Zeocin selection. Median fluorescence indicated by black dots. Dashed line indicates approximate cutoff of no fluorescence, as determined by wild-type HEK293T cells. **(a)** Transfection of dCas9-KRAB-DNMT3A. **(b)** Transfection of dCas9-DNMT3L-DNMT3A. **(c)** Transfection of dCas9-DNMT3A-KRAB. **(d)** Transfection of dCas9-DNMT3L-KRAB. **(e)** Transfection of dCas9-DNMT3A-DNMT3L


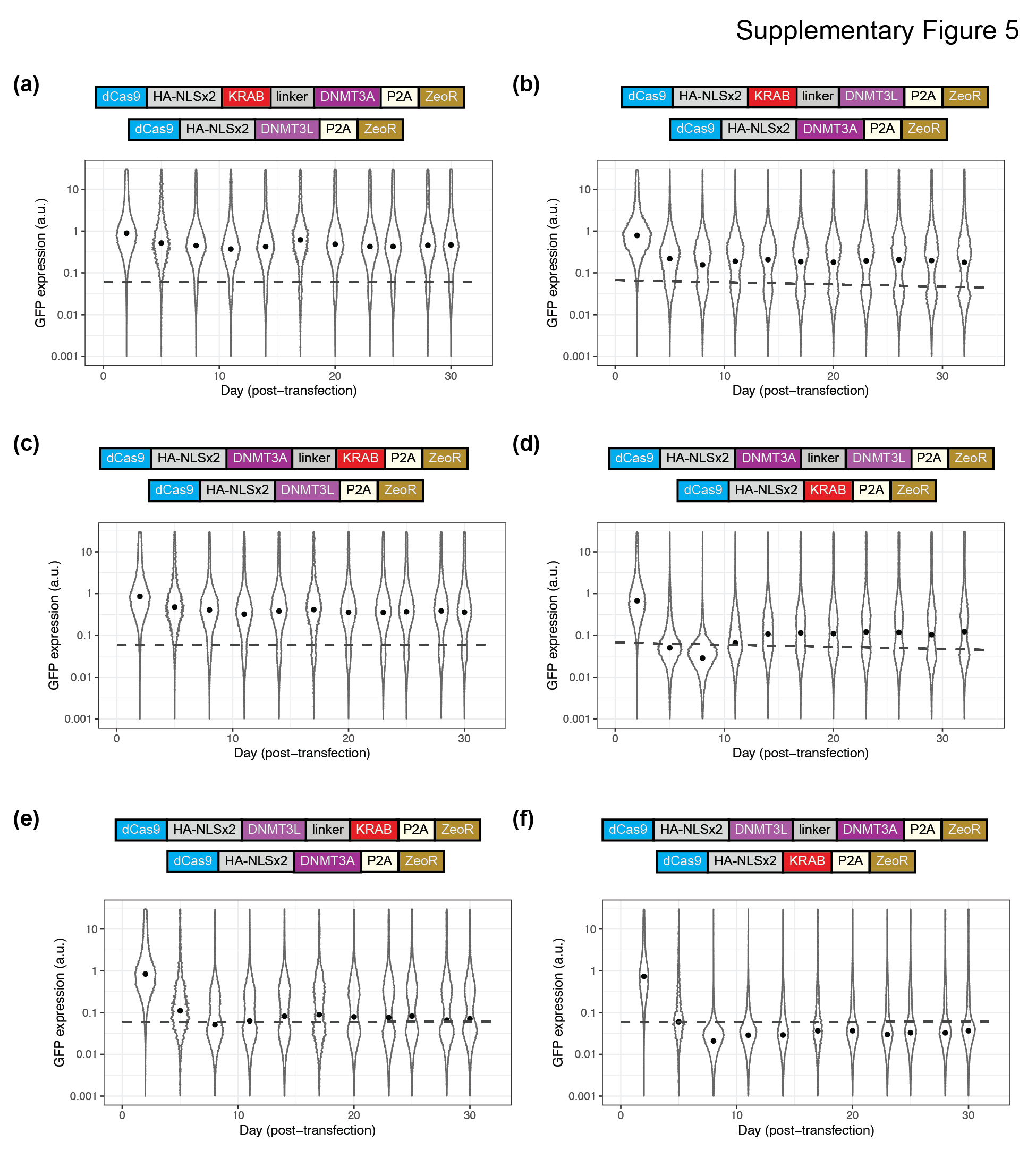


**Supplementary Figure 5 – combinations of double-effector and single effector constructs**

Representative violin plots of raw GFP fluorescence distributions over time following transient transfection of effector and Zeocin selection. Median fluorescence indicated by black dots. Dashed line indicates approximate cutoff of no fluorescence, as determined by wild-type HEK293T cells. **(a)** Co-transfection of dCas9-KRAB-DNMT3A and dCas9-DNMT3L. **(b)** Co-transfection of dCas9-KRAB-DNMT3L and dCas9-DNMT3A. **(c)** Co-transfection of dCas9-DNMT3A-KRAB and dCas9-DNMT3L. **(d)** Co-transfection of dCas9-DNMT3A-DNMT3L and dCas9-KRAB. **(e)** Co-transfection of dCas9-DNMT3L-KRAB and dCas9-DNMT3A. **(f)** Co-transfection of dCas9-DNMT3L-DNMT3A and dCas9-KRAB.


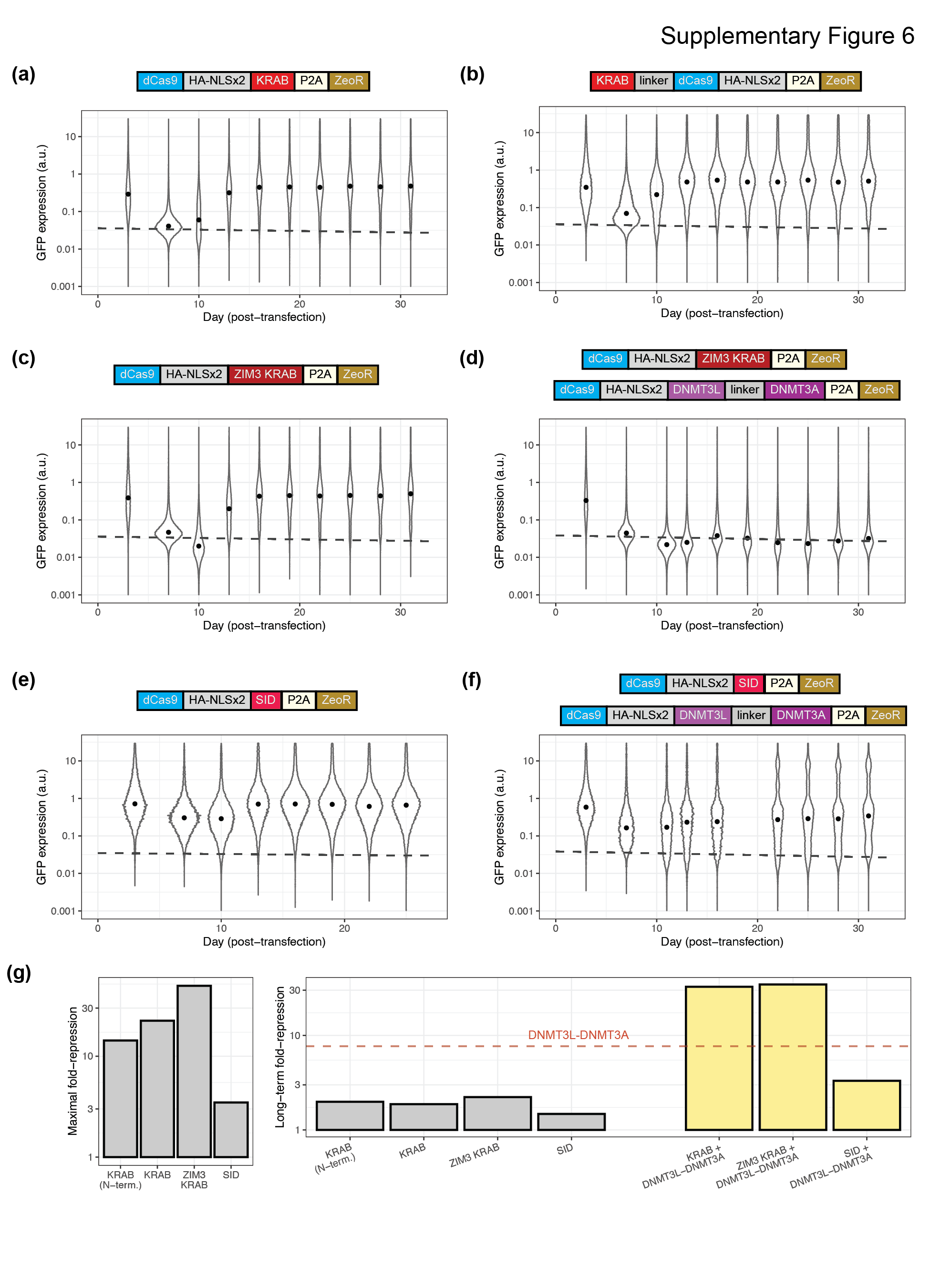


**Supplementary Figure 6 – characterization of temporary repressor variants**

**(a-f)** Representative violin plots of raw GFP fluorescence distributions over time following transient transfection of effector and Zeocin selection. Median fluorescence indicated by black dots. Dashed line indicates approximate cutoff of no fluorescence, as determined by wild-type HEK293T cells. **(a)** Transfection of dCas9-KRAB. **(b)** Transfection of KRAB-dCas9. **(c)** Transfection of dCas9-ZIM3 KRAB. **(d)** Co-transfection of dCas9-ZIM3 KRAB and dCas9-DNMT3L-DNMT3A. **(e)** Transfection of dCas9-SID. **(f)** Co-transfection of dCas9-SID and dCas9-DNMT3L-DNMT3A. **(g)** Left: characterization of the mean maximal fold-change observed for each timecourse across conditions. Right: characterization of long-term silencing abilities of repressors alone (left, gray) and in combination with dCas9-DNMT3L-DNMT3A (right, yellow). Red dashed line indicates silencing ability of dCas9-DNMT3L-DNMT3A alone.


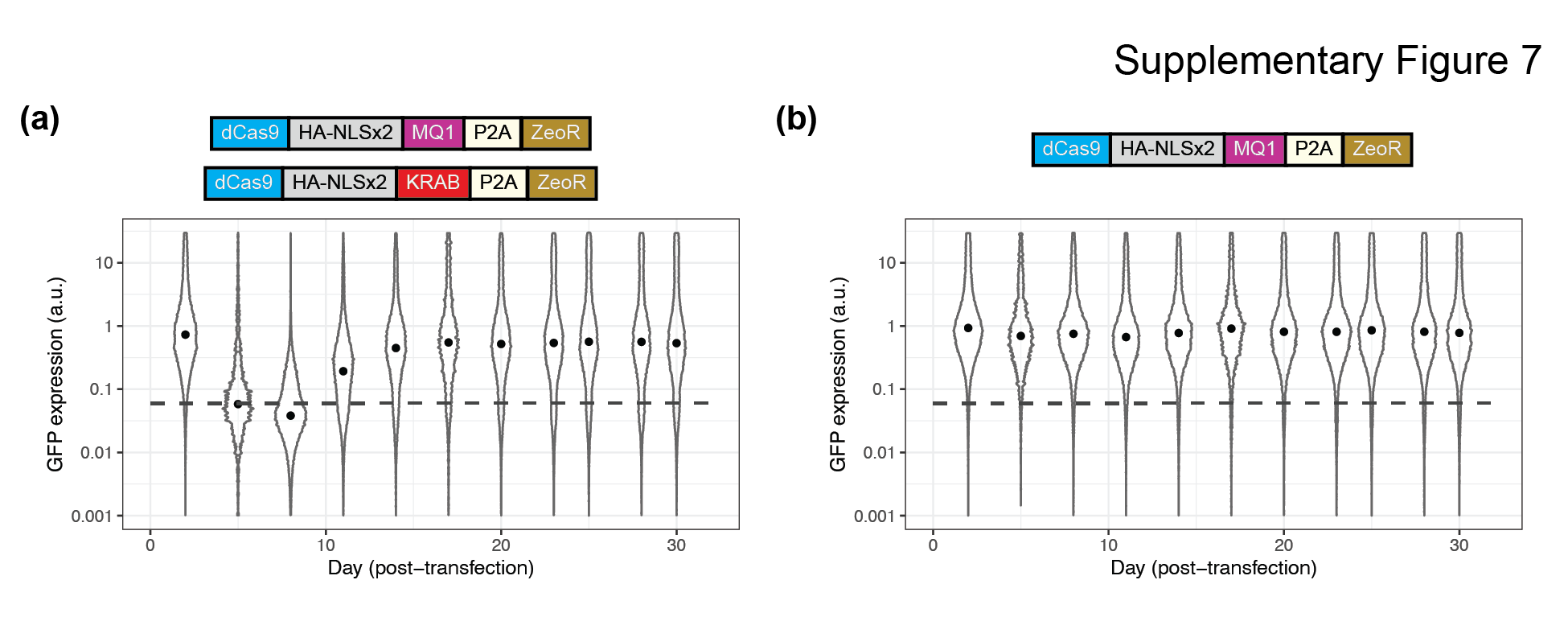


**Supplementary Figure 7 – characterization of dCas9-MQ1 silencing**

Representative violin plots of raw GFP fluorescence distributions over time following transient transfection of effector and Zeocin selection. Median fluorescence indicated by black dots. Dashed line indicates approximate cutoff of no fluorescence, as determined by wild-type HEK293T cells. **(a)** Co-transfection of dCas9-MQ1 and dCas9-KRAB. **(b)** Transfection of dCas9-MQ1.


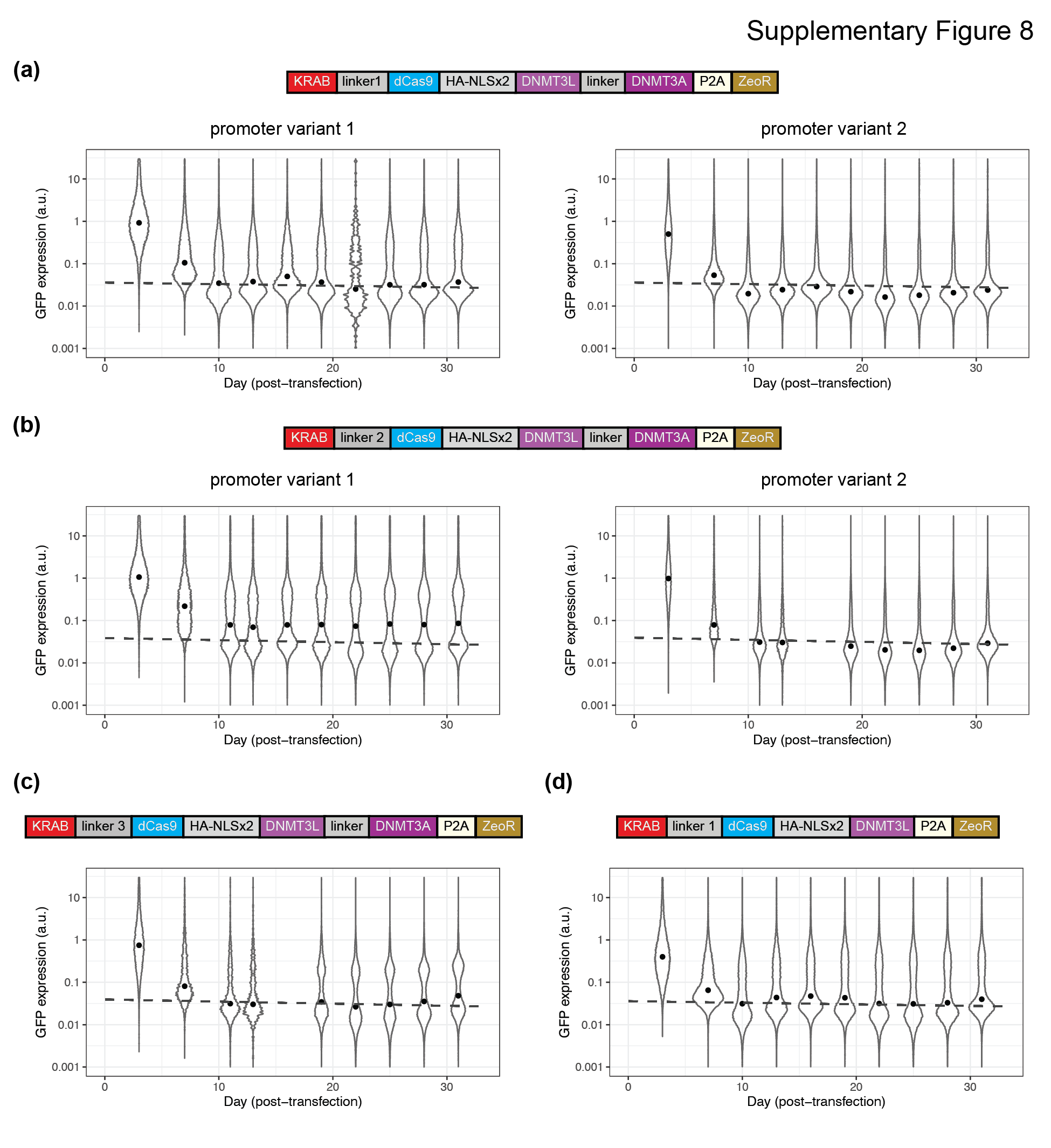


**Supplementary Figure 8 – characterization of KAL and KL effectors**

Representative violin plots of raw GFP fluorescence distributions over time following transient transfection of effector and Zeocin selection. Median fluorescence indicated by black dots. Dashed line indicates approximate cutoff of no fluorescence, as determined by wild-type HEK293T cells. **(a)** KRAB fused to dCas9-DNMT3L-DNMT3A with a short linker with two promoter variants. **(b)** KRAB fused to dCas9-DNMT3L-DNMT3A with a medium linker with two promoter variants. **(c)** KRAB fused to dCas9-DNMT3L-DNMT3A with a long linker. **(d)** KRAB fused to dCas9-DNMT3L with a short linker. **(c-d)** use promoter variant 2.


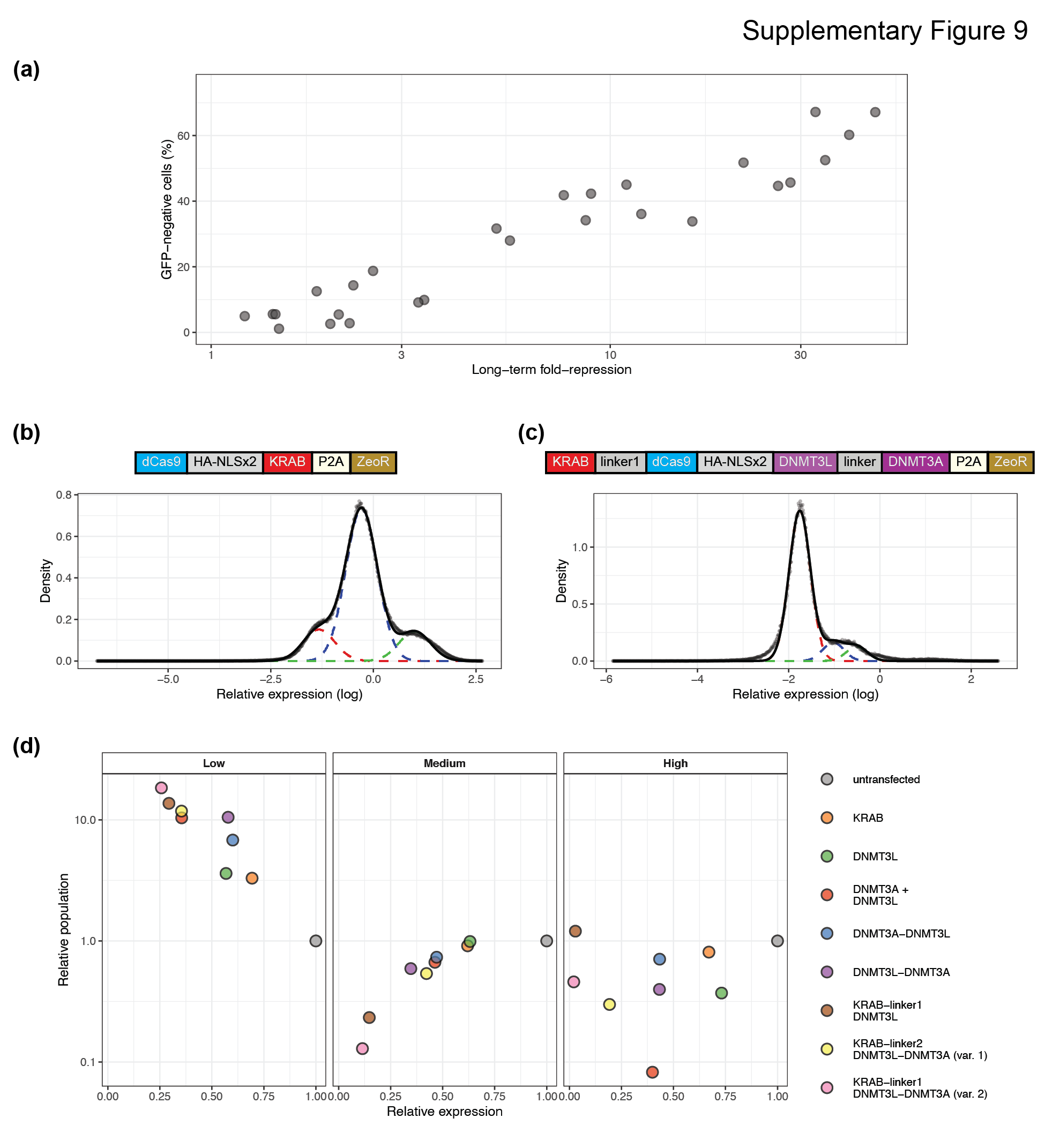


**Supplementary Figure 9 – population characterization of gene silencing**

**(a)** Plot of average GFP-negative cells 21 days or more post-transfection versus long-term fold-repression for each condition tested. **(b-c)** Representative triple-Gaussian fits of pooled cell populations 21 days or more post-transfection. Histograms of fluorescence intensity distribution shown as gray points. Colored dotted lines indicate each of the three Gaussians, with the summed fit distribution shown as a black line. **(b)** dCas9-KRAB condition. **(c)** dCas9-KAL condition. **(d)** Plots of parameters from fits as determined in **(b-c)**. The mean (relative expression) and area (relative population) from each Gaussian are normalized to the corresponding fit for untransfected reporter cells.
